# Gray Measure and Spatial Distribution Exploration of Local Emergency Resilience on Compound Disasters

**DOI:** 10.3390/ijerph191711071

**Published:** 2022-09-04

**Authors:** Feng Wu, Wanqiang Xu, Yue Tang, Yanwei Zhang, Chaoran Lin

**Affiliations:** 1 College of Public Administration, Huazhong University of Science and Technology, Wuhan 430074, China; 2 School of Public Administration, China University of Geosciences, Wuhan 430074, China; 3 College of Computer Science and Technology, Harbin Engineering University, Harbin 150001, China; 4 School of Economics and Management, Harbin Engineering University, Harbin 150001, China

**Keywords:** disaster management, emergency resilience evaluation, grey measure, spatial distribution, RAGA-PPM, Moran’s Index

## Abstract

The complexity and uncertainty of compound disasters highlight the significance of local emergency resilience. This paper puts forward a framework, including the Projection Pursuit Model based on Real-coded Accelerating Genetic Algorithm and the Moran’s Index (Moran’s I), to measure the local emergency resilience and analyze its spatial distribution. An empirical test is conducted with the case of Hubei Province, China. The results show that: (1) the measurement indices related to infrastructure, material reserves, and resource allocation have a larger weight, while those related to personnel and their practice have a smaller weight. (2) The measurement value of local emergency resilience of sub-provincial regions in Hubei Province is vital in the eastern and weak in the western, and there are apparent east-west segmentation and north-south aggregation characteristics. (3) Although the sub-provincial regions do not show significant spatial correlation, the eastern regions centered on Wuhan are negatively correlated, and the western regions are positively correlated. Furthermore, this study provides theories and methods for local emergency resilience evaluation and spatial correlation exploration, and it has specific guidance recommendations for optimizing local emergency management resource allocation and improving local emergency resilience.

## 1. Introduction

In the past decades, the rising frequency and severity of disasters have posed a severe threat to human society [1]. The hazard-inducing factors, evolutionary mechanisms, and hazardous forms of disasters have changed significantly owing to human society’s digitization, networking, and urbanization [2]. Compound disasters, which are highly complex and can involve different types of disasters [3], pose complex coordination and recovery challenges [4], and bring long-term devastation and shocks [5], have become the primary manifestation mode of disaster events and means the disaster prevention at the grassroots faces difficult situations. For example, in China, according to statistics, 107 million people were affected by natural disasters and a direct economic loss of 334.02 billion yuan in 2021.

Emergency management is a systematic work and is part of public management. The safe external environment for the general public can be regarded as a public product, and the participants who maintain the external environment are public management subjects. Local emergency management is the basis of disaster preparation and response. The local emergency management subjects under the vision of collaborative governance consist of public managers, social organizations, and hazard-bearing bodies. Therefore, to cope with the challenges of compound disasters, it is indispensable to enhance the resilience of the local emergency system [6]. For the local government, emergency resilience as a policy idea means providing better public products and services with less resource input [7]. Requirements for normalized emergency management have expanded the connotation of local emergency resilience from the dimensions of subjects’ practice activities and spatial effect. On the one hand, the learning ability [8] and adaptability [9] are attached to local emergency resilience, except for the resistance before and recovery after the disaster; local emergency resilience contains the capability of related subjects to overcome the negative effects of disasters, as well as the capacity of the disaster-bearing bodies to bear the losses. On the other hand, the carrier of the local emergency system is evolving into a systematic network instead of isolated cities or communities due to the complexity of disaster and the technicalization of disaster management [10]; the concept of local emergency resilience management has become more specific and holistic.

Compound features of the disaster and the requirements of normalized emergency management highlight the significance of emergency resilience enhancement, especially local emergency resilience. However, the conception of local emergency resilience under the new situation is still unclear. Moreover, for the complexity [11] and uncertainty [1] of compound disaster, could the local emergency resilience be measured with limited information, and how could it be applied? Does the local resilience have spatial correlations under the background of cross-regional and cross-sector cooperation? These are urgent issues to be analyzed for enhancing local emergency resilience and are the main focus of this study. Given this, we try to build a framework to measure local emergency resilience and explore its spatial distribution after explaining the concept of local emergency resilience in compound disasters.

The rest of this paper is organized as follows. Section 2 presents the related works of the core task of this study. Section 3 describes this study’s analytical framework and variable and presents the main methods, Projection Pursuit Model based on Real-coded Accelerating Genetic Algorithm (RAGA-PPM) and Moran’s Index (Moran’s I), applied in the empirical part. Section 4 describes the experiment based on the case of Hubei Province, China. Section 5 summarizes this study’s main conclusions and discusses the takeaways for practice.

## 2. Literature Review

This section investigates the previous related research of this study’s core concept and core content, including two separate sub-sections.

### 2.1. Concept Background of Local Emergency Resilience

The term resilience comes from the Latin word “resilio” [12], and describes the stability of materials and their resistance to external shocks in applied sciences [13]. Engineering, ecological, and social-ecological resilience are the theoretical development stages of resilience [14]. In the 1960s, it entered the field of ecology and was defined as “the magnitude of the disturbance that can be absorbed before the system changes its structure” [15,16]. In the 1980s, it was introduced into disaster management [13], and the early research focused on social vulnerability; since the 1990s, scholars realized that resilience should be more concerned with whether a society responds in time and recovers quickly after disasters [17]. Individual, community, and national resilience are the three levels in the relevant literature [18]; community and national resilience are regarded as social resilience in most studies [19].

Resilience in disaster risk management and emergency contains all the levels of resilience. There are four representative views to explain the concept of resilience in emergency management: the first holds that it is the recovery ability of human society’s infrastructure in external disturbances and contains the elements of institutional change, resource availability, economic structure, and population change [20]. The second view holds is that resilience is the amount of disturbance a social system can absorb and remain in the same state, and the degree to which the system is capable of self-organization [12]. The third points out that resilience could be examined systematically, including the layers of resistible capacity to external disturbance, institutional and organizational inertia and change, and adaptive capacity [21]. The fourth takes resilience (the social and ecological vulnerability) as the capacity to cope with uncertainty and surprise by mobilizing diverse sources of resilience [22]. We contend that the core content of resilience is the capability to adapt to external disturbances, maintain system balance, and show dynamic learning in disasters, and self-organization in chaos, which should be systematic and dynamic [2], by synthesizing these views.

### 2.2. Research on Local Emergency Resilience

The Rockefeller Foundation defined urban resilience as “the capacity of individuals, communities, institutions, businesses, and systems within a city to survive, adapt, and grow no matter what kinds of chronic stresses and acute shocks they experience” [23], which is widely used worldwide. Researchers have also defined local emergency resilience from different dimensions [24]. For compound disasters, local emergency resilience could be explained from two perspectives. The management subjects, mainly the local government and related apartments and organizations, address more adaptability in the dynamic situation. Meanwgile, the management objects, mainly including the general public and the infrastructure, focus more on the capacity to resist the disturbance and restore the system balance.

Researchers have conducted extensive research on different areas involved in local resilience, in which community-level [25,26,27,28,29,30,31,32,33,34,35] and urban-level [14,36,37,38,39] are the most concerning issues. Community resilience denotes a community’s capacity to lead itself to overcome changes and crises [33]; related research shows that emergency management at the local scale-municipal government level and community level are interdependent. Networks based on social capital [28] could improve the community’s emergency resilience [25]; thus, enhancing the community’s resilience to disasters is the primary goal of disaster management [26]. Disaster preparedness [27] and preparedness education [29,32] are national priorities [29], for which the policy is usually implemented by local departments [34,35]. Infrastructure [30,40], resources [33,36,41], as well as planning and warning communication [33,35] are indispensable elements of community resilience in disasters. For cross-regional disaster prevention, neighbors might function as resources for disaster preparedness [39], and cross-regional organizational relations could enhance resilience in each hierarchical and horizontal emergency management network [42]. Some researchers focus the issues of measurement [26,43], evaluation [38,44,45], and assessment [17] on local emergency resilience; these studies provide a set of indices and methodologies for local emergency resilience estimate under the compound disaster circumstance. Moreover, discussions on the resilient city or city resilience [46,47,48] also have positive significance in understanding and enhancing local emergency resilience.

The current research, however, is insufficient in establishing the evaluation index and methodology of local emergency resilience to compound disasters, and the spatial distribution of the resilience is still unclear.

## 3. Research Design

This study followed a descriptive correlation design. First, we put forward the analytical framework for measuring local emergency resilience to compound disasters. Then, we prepare the variables for local emergency resilience assessment by establishing the concept model of local emergency resilience from multiple dimensions. Based on these, a combined approach is put forward and described, including the local emergency resilience evaluation and the spatial correlation exploration methods.

### 3.1. Analytical Framework

The measurement of local emergency resilience is “cognitive uncertainty” of the lack of objective data and mature index and the uncertainty of compound disasters. At the same time, adequate information is the basis of local emergency resilience evaluation according to the principle of information cognition. To solve these problems of fewer data and uncertain, professor Deng put forward the Grey System Theory (shortened to Grey Theory) [49], which addressed the importance of extracting more valuable unknown information through the development of limited existing known information, and then revealing the overall operation and evolution mechanism of the system [50]. Limited data and uncertainty of data information are the dual characteristics of local emergency resilience measurements of compound disasters. The systematic attribute of disaster resilience [51], cross-regional [52] and cross-sector [30,53] cooperation networks, and the importance of strengthening resilience in mountainous [35,54,55] and rural [28,56,57] areas provides a theoretical basis for the existence of spatial correlation of local emergency resilience.

**Figure 1:**
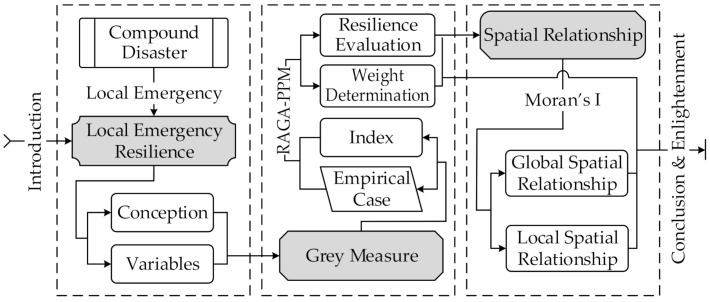
The analytical framework of this study.

Given this, we construct a grey analysis framework to evaluate the local emergency resilience and make an exploratory analysis of its spatial correlation. This framework consists of three modules (as shown in Figure 1). The first is variable preparation: prepare the main variables of local emergency resilience measurements of compound disasters to pave the way for constructing the measurement index based on explaining the concept of local emergency resilience. The second is the measurement of local emergency resilience based on empirical cases. This part builds a personalized measurement index and introduces the Real-coded Accelerating Genetic Algorithm Projection Pursuit Model (RAGA-PPM) to calculate the index weights and each region’s local emergency resilience. The third is the spatial distribution analysis of local emergency resilience, aiming to explore the global and local spatial relationships of the local emergency resilience between different regions using Moran’s Index (Moran’s I).

### 3.2. Variable Preparation

Local emergency management aims to keep the dynamic balance between the management subject and object and the external environment, that is, the balance in the process of management or governance. This balance synchronizes with disaster-inducing factors and the urgency of disaster prevention, making the balance’s frame of reference dynamic. Under normalized emergency management, the resistance and recovery capability of the local emergency system is the core of local emergency resilience, which have homologous heterogeneity with the division of local disaster management into three stages [32] or four stages [58]. At the same time, applying technical methods in disaster prevention puts higher requirements for learning ability and adaptability of the management subjects and objects, highlighting the importance of the dynamic adaptability of local emergency resilience. In addition, reducing compound disasters is a type of local governance [59]; full participation and collaboration of related subjects are necessary to improve governance effectiveness. In this governance process, local government plays the role of leader and coordinator and aims to realize the “1 + 1 > 2” governance efficiency through collaborating subjects and resources. Therefore, the system’s collaboration capacity is critical to local emergency resilience.

**Figure 2:**
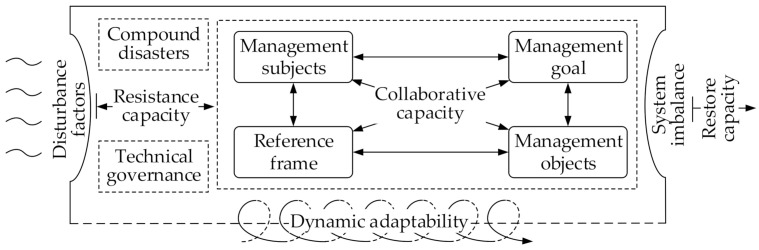
Concept model of local emergency resilience.

To summarize, we classify local emergency resilience into four dimensions: resistance capacity, restore capacity, dynamic adaptability, and collaborative capacity. The concept model of local emergency resilience in this study is shown in Figure 2.

### 3.3. Methodologies

The core of local emergency resilience measurement is calculating the index weight and the value of local emergency resilience. On this basis, the spatial correlations can be explored.

#### 3.3.1. Grey Measure of Local Emergency Resilience

Considering the objectivity of the results of the grey measure, we take the Projection Pursuit Model (PPM), a statistical method for analyzing high-dimensional non-normal and nonlinear data [60], to evaluate the local emergency resilience of compound disasters. PPM’s principle is to pursue the characteristic projection of high-dimensional observation data in low-dimensional format to analyze high-dimensional observation data [61]. When used for index weighting, PPM has better accuracy, robustness, and anti-interference than the Analytic Hierarchy Process (AHP) and Entropy Method [62]. When analyzing the local emergency resilience, PPM can calculate the index weight and the value of emergency resilience with the homologous small sample cross-section data, which can avoid the endogenous deviation caused by the data source. The main steps of PPM are:

1. Data standardization. If the sample size of the measurement object is m and the number of indices is n, the sample set is $X=\left\{x^{*}\left(i,j\right)|i=1,2,\ldots ,m;j=1,2,\ldots ,n\right\}$. To eliminate the interference of inconsistent index dimensions, the data set is standardized:
  (1) $$x\left(i,j\right)=\left\{\begin{array}{l} \frac{x^{*}\left(i,j\right)-x_{min}(j)}{x_{max}(j)-x_{min}(j)}(positive\;indicator)\\ \frac{x_{max}(j)-x^{*}\left(i,j\right)}{x_{max}(j)-x_{min}(j)}(negative\;indicator) \end{array}\right.$$
2. Construct the projection index function F(a). Optimal projection value of PPM could be seen as the index weights, the optimal projection scheme $a=\left\{a\left(1\right),a\left(2\right),\ldots ,\;a\left(n\right)\right\}$ is obtained from the projection of n-dimensional data $\left\{x\left(i,j\right)|j=1,2,\ldots ,n\right\}$ into the low-dimensional subspace. So, the projection value can be obtained as:
  (2) $$y(i)=\sum_{j=1}^{n}a(j)\times x(i,j),i=1,2,\ldots m$$
  Define the standard deviation of projection value y(i) as:
  $$S_{y}=\sqrt{\left(\sum_{i=1}^{m}{\left(y(i)-E(y)\right)}^{2}\right)/(m-1)}$$
  and the local density is:
  $$D_{y}=\sum_{i=1}^{m}\sum_{j=1}^{n}\left(R-r(i,j)\right)\times u\left(R-r(i,j)\right)$$
  then the projection index function is: $F\left(a\right)=S_{y}\times D_{y}$.
3. Projection function optimization. Since the value of the projection index function $F\left(a\right)$ will change with the change of the projection direction a, the estimation of the optimal projection direction can solve the maximization problem of the projection index function; that is, the maximization objective function is:
  (3) $$F{\left(a\right)}_{max}=S_{y}\times D_{y}$$
  (4) $$s.t.\sum_{j=1}^{n}a{\left(j\right)}^{2}=1$$
4. Obtain the projection value. The best projection direction $a^{*}$ is substituted into Equation (2) to obtain the best projection value $y^{*}(i)$, which is the measurement index’s weight or the value of the measurement object’s local emergency resilience.

The core of PPM is the optimization of projection function, which is to solve the optimal projection direction and the corresponding optimal projection value. The implementation of the PPM is simpler if the projection index function is optimized by using the Real-Coded Accelerate Genetic Algorithm (RAGA) [63]. It can be used to optimize the projection function, which can overcome the shortcomings of a large amount of calculation, premature convergence, and easily fall into the local optimum of the commonly used nonlinear optimization algorithms, like the Standard Genetic Algorithm (SGA) and Simulated Annealing (SA). Moreover, it can better overcome the Hamming–Cliff problem of the binary algorithm and has good optimization performance and simple coding [64]. The main steps of RAGA are:

1. Model Parameter Coding. Linear transformation of sample data as follows:
  (5) x(j)=a(j)+z(j)×(b(j)−a(j)), j=1,2,…,n

According to the Equation (3), F is the objective function to be optimized and n is the number of variables to be optimized. The real number $\left[a(j),b(j)\right]$ corresponding to the j-th variable x(j) to be optimized on interval $\left[0,\;1\right]$ on interval z(j) is calculated by Formula (5). As the genetic gene of RAGA, chromosome H can be obtained by orderly combination of all genetic genes. Equation (8) is used to calculate the real number z(j) corresponding to the j-th variable x(j) to be optimized in interval $\left[a(j),b(j)\right]$ on interval $\left[0,\;1\right]$, which is used as the genetic gene of RAGA. Chromosome $\left(z(1),z(1),\ldots ,z(n)\right)$ can be obtained by combining all genetic genes orderly.

2. Define the initial parent group. Assuming that the number of parent groups is s, the random number $\left\{u(i,j)|(i=1,2,\ldots ,n;j=1,2,\ldots ,n\right\}$ of s groups of interval $\left[0,1\right]$ with capacity n can be obtained, and then u(j,i) is the survivability value z(j,i) of the initial parent group. Substituting it into Equation (5), the priority change value x(j,i) can be obtained.
3. Establish fitness evaluation function $eval\left(z(j,i)\right)$ based on chromosome sequence. The probability of each chromosome in the population is set to ensure that the probability of chromosome replication is proportional to its fitness. Assuming that parameter α∈(0,1) is given, the replication function is:
  (6) $$eval(z(j,i))=\alpha {\left(1-\alpha \right)}^{1-i},\;i=1,2,\ldots ,N$$
4. Select the next generation. The chromosome replication is iterative by Roulette Algorithm. After each iteration, a group of new chromosomes is generated. After iteration N, the next generation population is marked as $\left\{z_{1}(j,i)|j=1,2,\ldots ,n\right\}$. The cumulative probability $q_{i}(i=1,2,\ldots ,N)$ calculated by the individual value z(j,i) of each chromosome is:
  (7) $$\left\{\begin{array}{l} q_{0}=0\\ q_{i}=\sum_{j=1}^{i}eval(z(j,i)),j=1,2,\ldots ,n;i=1,2,\ldots ,N \end{array}\right.$$
  Take the random number r in interval $\left[0,\;q_{i}\right]$, if $q_{i-1}<r\leq q_{i}$, then take the i-th chromosome z(j,i) to repeat the step 2 and step 3 N times to get the next N groups of replicated chromosomes.
5. Obtain the second generation by hybridizing the parent group. Define $P_{c}$ as the probability of cross-inheritance of the parent population, and if $r<P_{c}$, take z(j,i) as a parent. Donate the parent population as $z_{1}^{’}(j,i)$, $z_{2}^{’}(j,i)$, …, $z_{N}^{’}(j,i)$, and randomly paired as $\left(z_{1}^{’}(j,i),z_{2}^{’}(j,i)\right)$, $\left(z_{3}^{’}(j,i),z_{4}^{’}(j,i)\right)$, $\left(z_{N-1}^{’}(j,i),z_{N}^{’}(j,i)\right)$, and the second generation group is calculated as $\left\{z_{2}(j,i)|j=1,2,\ldots ,n;i=1,2,\ldots ,N\right\}$.
6. Solve a new mutation population. It is assumed that $P_{m}$ is the probability of variation population, and that the variation is similar to Step 5. When $r<P_{m}$, chromosome $z_{3}^{’}(j,i)$ is the parent population with variation. The variation in any direction d in n-dimensional space is denoted as: $A=z_{3}^{’}(j,i)+M\times d$, i=1,2,…,n, M being the random number on (0,1). Repeating this step can solve the new generation of population after mutation: $\left\{z_{3}(j,i)|j=1,2,\ldots ,n;i=1,2,\ldots ,N\right\}$.
7. Accelerate loop evolution iteration. Sort the 3N progeny populations according to the value of fitness function, take the first N−K individuals with strong viability as the new parent population, and start the iteration from step 3. Turn to step 1 again to eliminate the impact of too many iterations on the optimization ability of the algorithm, and accelerate this cycle. End the operation when the number of iterations or the optimal objective function is the set value. The optimal population solved is the optimal projection direction $a^{*}$, which can be substituted into Equation (2) to obtain the optimal projection value.

**Figure 3:**
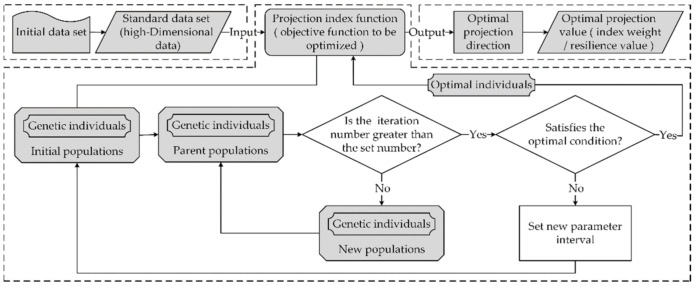
Technology roadmap of RAGA-PPM.

The technology roadmap of RAGA-PPM in local emergency resilience evaluation in this study is as shown in Figure 3:

#### 3.3.2. Spatial Distribution of Local Emergency Resilience

Take the Exploratory Spatial Data Analysis to explore the spatial relationship of local emergency resilience. This method can reveal the spatial heterogeneity and dependence of different regions by analyzing the spatial correlation and spatial variation of data [65]. This paper primarily analyzes the global and local correlation of local emergency resilience governance ability. The Global Moran’s Index calculates the global correlation, and the formula is:

(8) $$I=\frac{S}{\sum_{i}\sum_{j}w_{ij}}\frac{\sum_{i}\sum_{j}w_{ij}\left(x_{i}-\bar{x}\right)\left(x_{j}-\bar{x}\right)}{\sum_{i}{\left(x_{i}-\bar{x}\right)}^{2}}$$

S is the number of regions, $x_{i}$ is the value of emergency resilience in the i region, $\bar{x}$ is the average value of emergency resilience in all regions, $w_{ij}$ is the spatial weight of the i and j regions, and $\sum_{i}\sum_{j}w_{ij}$ is the aggregation of all spatial weights.

Take the Local Moran’s Index to analyze the local correlation of local emergency resilience, the formula is:

(9) $$I_{i}=\frac{S(x_{i}-\bar{x})\sum_{j}w_{ij}(x_{j}-\bar{x})}{\sum_{i}{\left(x_{i}-\bar{x}\right)}^{2}}$$

The value ranges of I and $I_{i}$ are [−1, 1]. When the value is less than 0, there is a negative spatial correlation; when the value is greater than 0, there is a positive correlation; when the value is equal to 0, there is no spatial correlation.

## 4. Case Analysis of Hubei Province

We take the Hubei Province as a case and deploy the framework proposed above to measure its local emergency resilience and explore the spatial correlations. Before the measurement, we conducted the comparison experiment using Entropy-TOPSIS to calculate the weight of the indices and the value of local emergency resilience. It is believed that the RAGA-PPM is more reliable for comparing the results obtained by these two approaches and consulting the relevant management personnel of the emergency management department of Hubei Province.

### 4.1. Case Selection, Index Construction and Data Collection

#### 4.1.1. Case Selection

We take 17 sub-provincial administration units in Hubei Province (including autonomous prefectures, county-level cities under the provincial jurisdiction, and forestry districts) as empirical cases, and the reasons are as follows: firstly, to ensure the horizontal comparability and data availability of the case in this study. Because the difference in disaster types at the provincial level is too significant, it is hard to make a horizontal comparison of different regions; the difference between county administrative units is too small and lacks objective data. Secondly, Hubei Province has good representativeness, which can ensure the practical reference value of the results. Regarding geographical location, Hubei Province is located on the boundary between the east and west of China and the north and south of geography, combining multiple disaster attributes. Regarding climate and topography, Hubei Province is high in the west and low in the east, with noticeable differences between the east and the west; most sub-provincial administration regions belong to semi-tropical monsoon climate and plateau alpine climate, which are typical types in China. Regarding regional development, Hubei Province has large central cities and remote mountainous areas, with significant differences in population, economy, transportation and culture. These attributes of Hubei Province form multiple internal control groups for local emergency resilience analysis. Moreover, as the first area to detect and deal with COVID-19 worldwide, its early emergency response can be used as an external reference to test the property of the empirical results of this study.

#### 4.1.2. Index Construction

Based on the disaster characteristics of Hubei Province, the conceptual model of local emergency resilience aforementioned, and referring to the related indices [16,62], we construct the index from the dimension of resistance capacity, restorative capacity, collaborative capacity, and dynamic adaptability (Table 1).

Resistance capability focuses on the local emergency system’s population, equipment, and environmental status before disasters and the preventative and control-based ability in the early stage. Resistance capability focus on the local emergency system’s population, equipment, and environmental status before disasters, as well as prevention and control ability in the early stages. Restore capacity focuses on post-disaster relief, reconstruction, rehabilitation capabilities, and the state of emergency supplies and emergency personnel. Collaborative capacity focuses on the capability of social participation, information sharing, resource allocation, and material transportation. Dynamic adaptability focuses on the ability to have knowledge of learning and application, normalize governance capability, improve disaster information perception and transmission ability, and the management efficiency of risk factors.

#### 4.1.3. Data Collection and Preprocessing

To present the current situation of local emergency resilience of sub-provincial regions in Hubei Province objectively, we use the cross-sectional data from 2021 to conduct the experiment. The data was obtained with the assistance of the Hubei Provincial Emergency Management Department, and the use of the data has been approved. The missing values of the original data (the number of psychological consultants in Huangshi and Shennongjia Forest District, amount of highway mileage in Xianning, the volume of safety education publicity in Jingmen, the value of rescue equipment in Xiangyang, and the number of reservists in Yichang) were supplemented by a comprehensive interpolation method based on Bayesian estimation. The method considers that the missing value to be interpolated is random, and it depends on the other observed values. By estimating the value to be interpolated and then adding different noises, multiple sets of optional interpolation values are formed, and the most suitable group is selected as the interpolation value according to specific rules.

### 4.2. Index Weighting and Resilience Measurement

#### 4.2.1. Index Weighting

**Figure 4:**
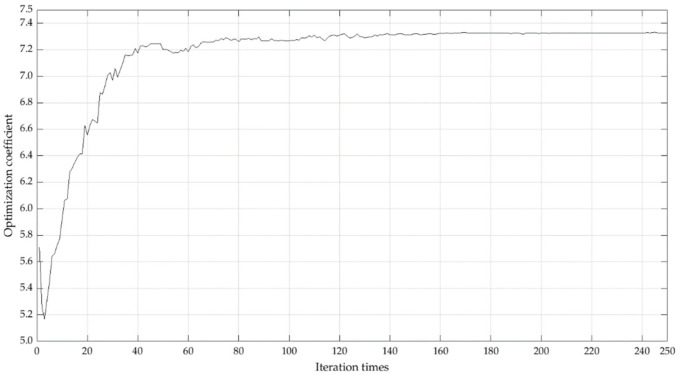
Iteration trend of the optimal projection value.

**Figure 5:**
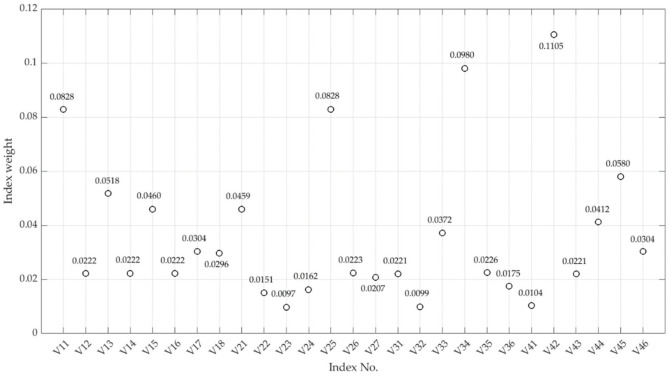
Optimal projection value of local emergency resilience.

Use MATLAB software to calculate the weight of the local emergency resilience measurement index of compound disasters according to the principles and steps of RAGA-PPM. Taking the index sequence of the standardized data matrix as the observation sequence, through exploratory experiment, we set the population size N=40, the crossover genetic probability $P_{c}=0.8$, the mutation genetic probability $P_{m}=0.2$, the number of optimization variables n=2, the random number for the mutation direction M=3, and iteration times are 250. The RAGA-PPM’s iteration trend of the optimal projection value of the evaluation index is shown in Figure 4:

It shows that when the iteration time is close to 140, the amplitude of the model gradually converges. When the iteration time is 160, the model tends to be constant, indicating that the calculation results are stable; the optimal projection value of each index can be obtained (as shown in Figure 5).

#### 4.2.2. Resilience Measurement

**Figure 6:**
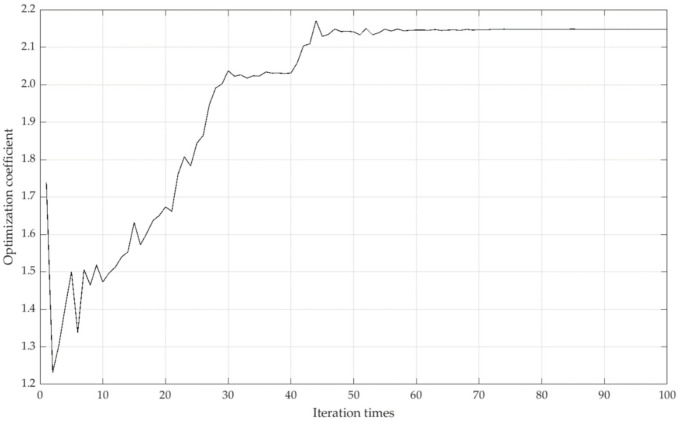
Iteration trend of optimal the projection value of local emergency resilience.

**Figure 7:**
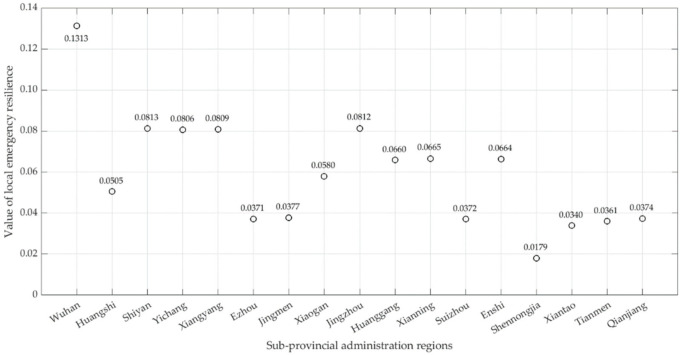
Value of the local emergency resilience.

Taking the regional sequence of the standardized data matrix as the observation sequence, we set the population size N=34, the crossover genetic probability $P_{c}=0.8$, the mutation genetic probability $P_{m}=0.2$, the number of optimization variables m=1, the random number required in the mutation direction M=2, and 100 iterations. The iteration trend of RAGA-PPM is shown in Figure 6:

It can be seen that when the iteration time is about 50, the amplitude of the model gradually converges. When the number of iterations is 60, the model tends to be constant, indicating that the calculation results are stable; the optimal projection value of the local emergency resilience of each region can be obtained (as shown in Figure 7).

**Figure 8:**
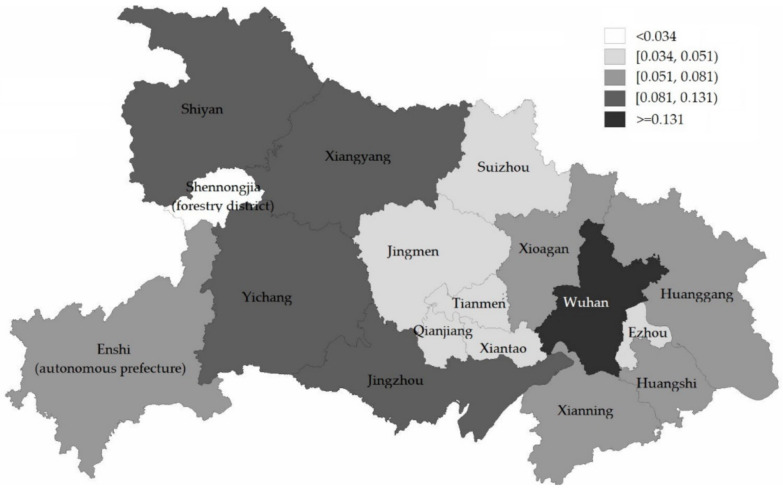
Classification of local emergency resilience across regions.

Make use of Jenks’ Best Natural Fracture Method [66] to classify local emergency resilience in different regions, including five levels (Figure 8). The first level is Wuhan, the emergency resilience value of which is more than 0.131 ($y_{i}\geq 0.131$). The second level includes Shiyan, Jingzhou, Xiangyang, and Yichang ($0.081\leq y_{i}<0.131$). The third level consists of the Xianning, Enshi autonomous prefectures, Huanggang, Xiaogan, and Huangshi ($0.051\leq y_{i}<0.081$). The fourth level includes Jingmen, Qianjiang, Ezhou, Suizhou, Tianmen, and Xiantaoc ($0.034\leq y_{i}<0.051$). The fifth level in Shennongjia forestry district ($y_{i}<0.034$).

**Figure 9:**
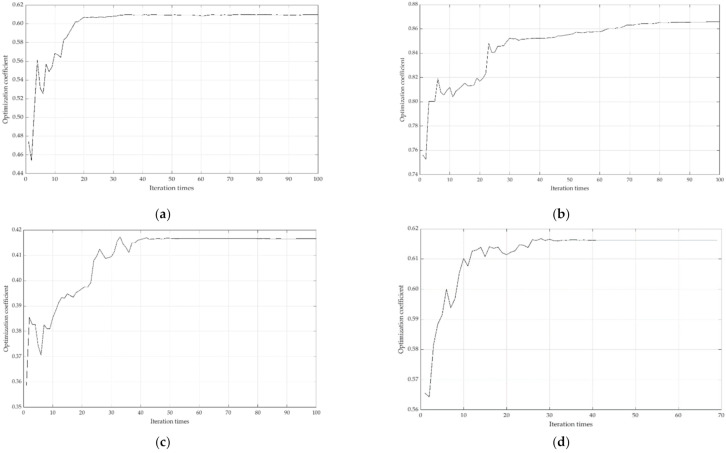
Iteration trend of RAGA-PPM of local emergency resilience in each dimension: (**a**) iteration trend of RAGA-PPM of resistance capacity; (**b**) iteration trend of RAGA-PPM of restore capacity; (**c**) iteration trend of RAGA-PPM of dynamic adaptability; (**d**) iteration trend of RAGA-PPM of collaborative capacity.

**Figure 10:**
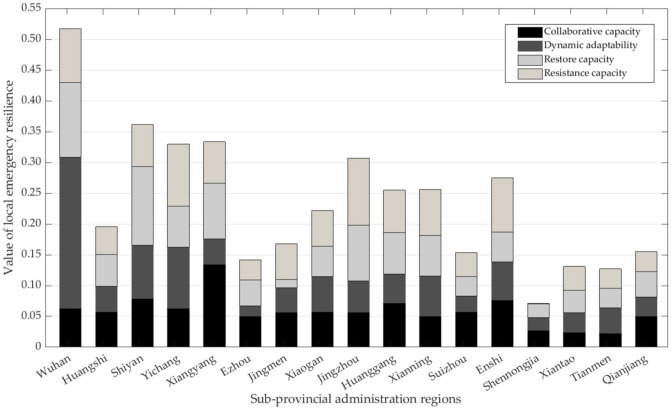
Value of local emergency resilience in four dimensions.

To further explore the composition of local emergency resilience in each dimension, we measure the local emergency resilience in the dimensions of resistance capacity (15 iterations), restore capacity (200 iterations), dynamic adaptability (400 iterations), and collaborative capacity (150 iterations) after the exploratory experiment, respectively. Set the population size N=34, the crossover genetic probability $P_{c}=0.8$, the mutation genetic probability $P_{m}=0.2$, the number of optimization variables m=1, the random number required in the mutation direction M=2, and at 100 iterations, the iteration trend of RAGA-PPM being shown in Figure 9:

Figure 9 shows that the optimization coefficients of the four dimensions of local emergency resilience calculation are stable under the above RAGA-PPM parameter setting, indicating that the results are reliable. Draw the histogram diagram of the value of local emergency resilience in four dimensions, as shown in Figure 10:

Figure 10 shows that the differences in resilience capacity, dynamic adaptability, and collaborative capacity in different regions are relatively minor, while the differences in restored capacity are significant. Wuhan, Yichang, Xianning, Suizhou, and Enshi have significant differences in each dimension of local emergency resilience within the group, while Huangshi, Shiyan, Huanggang, Xiantao, and Tianmen have minor differences. Wuhan has a weak collaborative capacity and the most robust dynamic adaptability, which is consistent with the characteristics of mega-central cities. Shennongjia forestry district has the weakest resistance capacity; this may be related to the region’s tiny economy, sparse population, and underdeveloped transport (rescue difficulties).

It should be noted that the value of the local emergency resilience is not equal to the weighted mean value of local emergency resilience of the four dimensions calculated, even though the data sources are the same. It reveals that for compound disaster management, local emergency resilience is not the arithmetic sum of each dimension’s resilience. That is also an essential difference between RAGA-PPM and AHP, Entropy, and Comprehensive Evaluation Method.

### 4.3. Resilience’s Spatial Distribution

#### 4.3.1. Global Spatial Relationship

**Figure 11:**
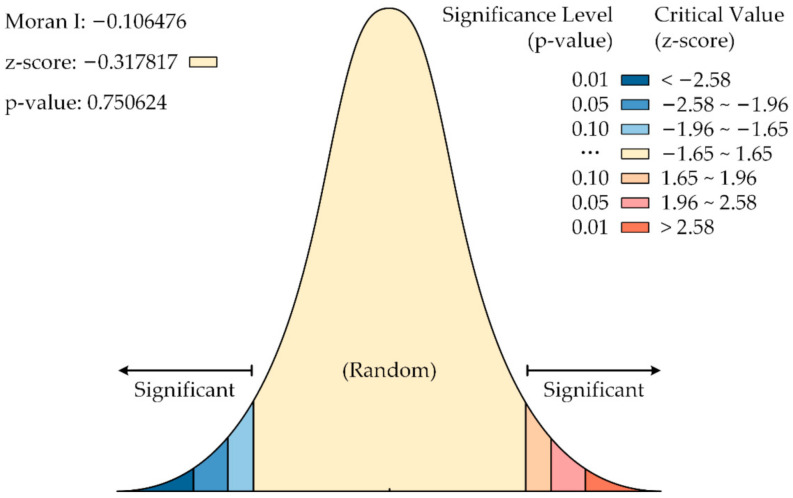
Global Moran’s Index report.

To explore the spatial correlations of local emergency resilience in Hubei Province, the Euclidean Distance Method is applied to calculate the Global Moran’s Index of local emergency resilience in sub-provincial administration regions; the Global Moran’s Index report is shown in Figure 11. In which the Global Moran’s Index I= −0.106476, the Expected Index is: 0.062500, the Variance is 0.019146, the z-score is −0.317817, and the *p*-value is 0.750624. These indicate an overall spatial negative correlation in local emergency resilience of the sub-provincial regions, but the correlation is not significant.

#### 4.3.2. Local Spatial Relationship

**Figure 12:**
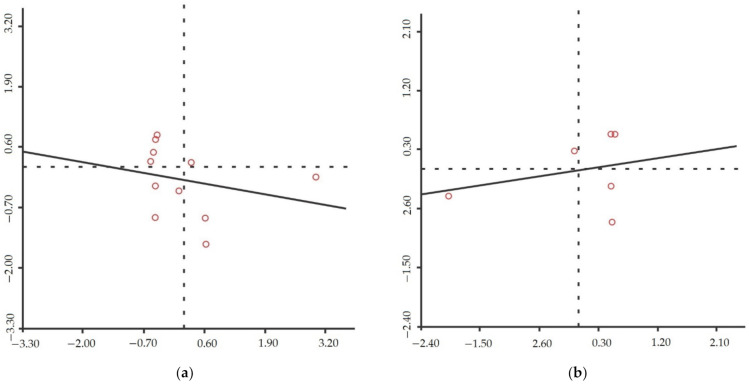
Scatter plots of the Local Moran’s Index: (**a**) scatter spot of Local Moran’s Index in the eastern regions; (**b**) scatter spot of Local Moran’s Index in the western regions.

Figure 8 shows that there is internal convergence in 11 eastern regions (including Wuhan, Suizhou, Jingmen, Tianmen, Qianjiang, Xiantao, Xiaogan, Huanggang, Ezhou, Huangshi, and Xianning, with Wuhan as the center) and six western regions (Shiyan, Xiangyang, Shennongjia Forest District, Yichang, Enshi Autonomous Prefecture, Jingzhou). Therefore, based on the global spatial correlation, we explore the local correlation of local emergency resilience in the eastern and western regions. The Local Moran’s Index in the eastern regions is −0.174, and in the western regions is 0.154. That is, the eastern regions’ local emergency resilience is negatively correlated, and the western regions’ is positively correlated. The scatter plots of the Local Moran’s Index are as shown in Figure 12:

The sub-provincial administrative regions corresponding to the points in the scatter plots are shown in Table 2:

In the eastern region, only Xiaogan has strong-strong aggregation, which is close to the origin point of the coordinate; weak–weak aggregation is Suizhou, Huangshi, and Ezhou; strong–weak (weak–strong) aggregation is Jingmen, Qianjiang, Tianmen, Xiantao, Xianning, Huanggang, and Wuhan. In the western region, strong–strong aggregation is Shiyan and Yichang; weak–weak aggregation is Shennongjia; strong–weak (weak–strong) aggregation is Enshi (located in the second quadrant), Xiangyang, and Jingzhou; strong–strong aggregation is Yichang and Shiyan. Overall, the number of cities in the first and third quadrants (positive correlation) is less than that in the second and fourth quadrants, consistent with the global spatial correlation. Given the lack of systematicity of emergency resilience in each dimension in response to compound disasters, this study does not analyze its spatial correlation.

### 4.4. Results Explanation

#### 4.4.1. Index Weight Distribution

The four most-weighted evaluation indices are “information sharing” (V42), “information transmission” (V34), “risk diffusion” (V11), and “public health restoration” (V26) (each of the four dimensions has one). Among them, V26, V34, and V42 belong to the infrastructure construction category, indicating that infrastructure construction and improvement is the key to strengthening local emergency resilience, especially for the information and medical infrastructure. V11 shows that the denser the population is, the more challenges confronted for local disaster management, which may become more complex and stereoscopic under compound disasters. The index group of the second largest weight is “land transport capacity” (V45), “disaster response” (V13), “rescue equipment support” (V15), “disaster relief material” (V22), and “resource distribution” (V44). This index group also involves all local emergency resilience dimensions, and the content of its index is mainly related to the storage, transportation, and deployment of disaster prevention and mitigation materials. It indicates that material reserves and traffic conditions are critical to local emergency resilience. In contrast, the weights of “reserve emergency force” (V24), risk resolution capability (V32), social participation capability (V41), standing emergency force (V23), post-disaster reconstruction capability (V25), and public economic recovery capability (V28) are the smallest. The reasons may be two-folw: the management of related index fields has been relatively perfect, such as fire protection, reserve mobilization, renovation of critical industries, emergency financial support, and the like. Second, the impact of the index on emergency resilience management is relatively weak, such as the limited participation ability of social rescue organizations in compound disaster management under “Meta-governance” [67].

#### 4.4.2. Spatial Distribution of the Resilience

Overall, the measurement results of emergency resilience in different sub-provincial administration regions are significantly different (the maximum value of Wuhan is seven times the minimum value of Shennongjia). Regions corresponding to the second level, third level, and fourth level of local emergency resilience measured value is characterized by east-west segmentation and north-south aggregation in geographical space; and there are three north-south distribution zones: “Shiyan–Xiangyang–Yichang–Jingzhou,” “Suizhou–Jingmen Tianmen–Qianjiang–Xiantao,” “Xiaogan–Huanggang–Huangshi–Xianning.” This feature coincides with the province’s eastern and western population, geography, economy, climate, and transportation differences. These factors also have an important impact on the generation, diffusion, and recovery of complex disasters.

#### 4.4.3. Spatial Correlation of the Resilience

The z-score and *p*-value of the global Moran index of local emergency resilience have not reached statistical significance, indicating that the overall distribution of local emergency resilience in Hubei Province has no significant spatial aggregation characteristics. However, the measured value of the Global Moran Index is negative, indicating that the overall spatial negative correlation, that is, adjacent regions, will have an inhibitory effect on each other’s emergency resilience. The value of the Local Moran Index shows that eleven regions in the eastern region are negatively correlated, and seven regions have strong-weak/weak-strong aggregation, making the eastern region negatively correlated. There is a positive spatial correlation among the six cities in the western region. Shiyan and Yichang have strong emergency resilience governance capabilities and show strong-strong aggregation, which makes the western region have a local positive correlation.

## 5. Conclusions and Discussion

### 5.1. Conclusions

The compositeness and complexity of disasters highlight the importance of enhancing local emergency resilience. As the local emergency system must adapt to external disturbances, maintain system balance, dynamic learning in disasters, and self-organization in chaos, it is indispensable to measure the local emergency resilience and explore its spatial distribution. Based on the characteristic of compound disasters, the requirement of local emergency management, and the embedding of technical governance, this paper explains the concept of local emergency resilience and then takes Hubei Province as an example to construct the measurement index and introduces the RAGA-PPM to calculate the index weight of the sub-provincial regions, and makes an exploratory analysis of its spatial characteristic. Comparing the empirical results with the actual situation, we harbor that the methodology in this paper can obtain objective results with high reliability for emergency management assessment problems with small samples and limited data.

Regarding the index weight, those related to infrastructure, material reserves, and resource allocation are larger, while those related to personnel and their practice are smaller. That indicates that local emergency resilience is mainly reflected in infrastructure construction, material reserves, and transportation capacity in response to compound disasters. As for the value of local emergency resilience, sub-provincial regions in Hubei Province are vital in the eastern, weak in the western, and extremely strong in central cities; there are apparent east-west segmentation and north-south aggregation characteristics. As the spatial distribution of local emergency resilience, although the sub-provincial regions do not show significant spatial correlation, the eastern regions centered on Wuhan are negatively correlated, and the western regions are positively correlated.

This study provides theory and method for local emergency resilience evaluation and spatial correlation exploration, and it has specific guidance recommendations for optimizing local emergency management resource allocation and improving local emergency resilience. The main limitation of this study is that the experimental data are limited to one province, and the spatial correlations are ignored between different provinces. Follow-up studies will try to expand the data source nationwide to explore the inter-provincial correlation of local emergency resilience. In addition, we only measured the local emergency resilience in one year, so it could be interesting to extend this study to the time evolution of local emergency resilience.

### 5.2. Discussion

For compound disaster management, the complexity, resource endowment, and spatial structure of the local emergency system are the critical internal factors for the advantages and disadvantages of the emergency resilience management capability in the four dimensions and also cause the resilience’s uneven spatial distribution. To strengthen the local emergency resilience and optimize the local emergency management system, the primary the sub-provincial regions need is established to overcome their shortcomings. Optimizing the spatial layout of governance resources is the premise and foundation of filling the capacity gap. These resources include tangible resources such as human resources, materials, transportation, and technology and intangible resources such as policies and collaboration platforms. Optimizing resource allocation can improve the resistance capacity by improving the complexity of the local emergency system and the restoration capacity, dynamic adaptability, and collaborative capacity by improving the self-organization ability of the system.

In addition, the provincial emergency system should adjust measures to local conditions and disasters to build a cooperation system suitable for regional development. The “core-periphery” theory [68] argues that the core and periphery are the essential spatial structural elements of the regional social system; they are interdependent and form a complete spatial system. The core area can organize and dominate the latter’s development in the regional development process. Wuhan and surrounding regions, including Qianjiang, Tianmen, Xiantao, and Ezhou, can be regarded as a “core-periphery” structure. Due to the absorption of surrounding resources by the core region and the homogeneity of disasters in this structure, a regionally integrated emergency system can be built. The governance efficiency can be transmitted into the peripheral regions by enhancing the polarization of the core region, and a two-way resource efficiency circulation mechanism can be established to strengthen the emergency resilience of the peripheral regions. By building a regionally integrated emergency system, the links between subsystems are closer, thus forming a higher-level system with higher complexity. This can improve the resistance capacity of the subsystem, and the regionally integrated emergency system also engages risk sharing.

Moreover, given the loose relations and scarce population, the disaster event would bring less damage than the eastern regions. However, they need more time to recover because of the weak personnel allocation, material reserves, and infrastructure construction. Strengthening regional cooperation, on the one hand, can improve the resistance capacity by increasing the system’s complexity; on the other hand, it may improve the utilization rate of resources by sharing the resources. Furthermore, it moves the disaster management gateway forward and reduces disasters’ impact by building an emergency mutual assistance mechanism. Specifically, the local regions should build local emergency mutual assistance platforms, smooth resource sharing channels, and establish emergency cooperation mechanisms.

## Figures and Tables

**Table 1:** Local emergency resilience evaluation index.

Dimension	Index (No.)	Index Measurement (Description)	Attribute ^1^
Resistance capacity	Risk diffusion V11	Population per square kilometer	N
	Disaster inhibition V12	Number of major disasters	N
	Disaster response V13	Number of emergency plans	P
	Disaster control V14	Direct economic losses from disasters (10,000 Yuan)	N
	Rescue equipment support V15	Value of emergency rescue equipment (10,000 Yuan)	P
	Disaster avoidance V16	Disaster shelter capacity (10,000 people)	P
	Ecological maintenance V17	Number of forest protectors per 10,000 people	P
Restore capacity	Personnel treatment V21	Number of hospital beds	P
	Disaster relief material V22	Value of disaster prevention material (10,000 Yuan)	P
	Standing emergency force V23	Number of firefighters	P
	Reserve emergency force V24	Number of reservists	P
	Post-disaster reconstruction V25	Local fiscal revenue (10,000 Yuan)	P
	Public health restoration V26	Basic medical insurance coverage (%)	P
	Public psychological repair V27	Number of psychological consultants	P
	Public economic recovery V28	Gross national product (10,000 Yuan)	P
Dynamic adaptability	Disaster prevention consciousness V31	Number of safety education publicity	P
	Disaster risk resolve V32	Number of safety production remediation	P
	Information perception V33	Number of disaster information officers	P
	Information transmission V34	Communication line coverage (%)	P
	Normalized management V35	Emergency management budget (10,000 Yuan)	P
	Disaster prevention exercise V36	Number of disaster prevention exercises	P
Collaborative capacity	Social participation V41	Number of civil emergency rescue personnel	P
	Information sharing V42	Mobile network coverage (%)	P
	Public opinion guidance V43	Information emergency investment (10,000 Yuan)	P
	Resource distribution V44	Stuff number of emergency management department	P
	Land transportation capacity V45	Highway mileage (km)	P
	Water transportation capacity V46	Inland waterway mileage (km)	P

^1^ “N” represents negative, “P” represents positive.

**Table 2:** Regions corresponding to the points in the scatter plots.

Quadrant	Eastern Regions	Western Regions
First	Xiaogan	Shiyan; Yichang
Second	Jingmen; Qianjiang; Tianmen; Xiantao	Enshi
Third	Suizhou; Huangshi; Ezhou	Shennongjia
Fourth	Xianning; Huanggang; Wuhan	Xiangyang; Jingzhou

## Data Availability

Not applicable.
